# Molecular profiling of pre- and post- 5-azacytidine myelodysplastic syndrome samples identifies predictors of response

**DOI:** 10.3389/fonc.2024.1438052

**Published:** 2024-09-23

**Authors:** Mónica del Rey González, Sohini Chakraborty, Jesús María Hernández-Sánchez, María Diez Campelo, Christopher Y. Park, Jesús María Hernández Rivas

**Affiliations:** ^1^ Institute for Biomedical Research of Salamanca (IBSAL), Institute of Cancer Molecular and Cellular Biology (IBMCC)-Centro de Investigación del Cáncer, Universidad de Salamanca, Salamanca, Spain; ^2^ Department of Pathology, New York University Grossman School of Medicine, New York, NY, United States; ^3^ Hematology Department, Hospital Universitario Salamanca, Salamanca, Spain

**Keywords:** myelodysplastic syndrome (MDS), hematopoietic stem/progenitor cells (HSPCs), mutations, gene expression, patient survival, prognosis

## Abstract

Treatment with the hypomethylating agent 5-azacytidine (AZA) increases survival in high-risk (HR) myelodysplastic syndrome (MDS) patients, but predicting patient response and overall survival remains challenging. To address these issues, we analyzed mutational and transcriptional profiles in CD34+ hematopoietic stem/progenitor cells (HSPCs) before and following AZA therapy in MDS patients. AZA treatment led to a greater reduction in the mutational burden in both blast and hematological responders than non-responders. Blast and hematological responders showed transcriptional evidence of pre-treatment enrichment for pathways such as oxidative phosphorylation, MYC targets, and mTORC1 signaling. While blast non-response was associated with TNFa signaling and leukemia stem cell signature, hematological non-response was associated with cell-cycle related pathways. AZA induced similar transcriptional responses in MDS patients regardless of response type. Comparison of blast responders and non-responders to normal controls, allowed us to generate a transcriptional classifier that could predict AZA response and survival. This classifier outperformed a previously developed gene signature in a second MDS patient cohort, but signatures of hematological responses were unable to predict survival. Overall, these studies characterize the molecular consequences of AZA treatment in MDS HSPCs and identify a potential tool for predicting AZA therapy responses and overall survival prior to initiation of therapy.

## 1 Background

Myelodysplastic syndromes (MDS) are clonal diseases arising in hematopoietic stem cells (HSCs) characterized by altered myeloid differentiation, peripheral blood cytopenia, and frequent accumulation of blasts (1, 2). While most patients experience progressive cytopenia, approximately 30% progress to acute myeloid leukemia (AML) (3). Hypomethylating agent (HMA) therapies including 5’-azacytidine (AZA) and decitabine (DAC) are among the few therapies available for patients ineligible for allogeneic stem cell transplantation, especially patients with high-risk myeloid neoplasms (4, 5).

AZA is an azanucleoside analog of cytidine approved for use in MDS patients in 2004 based on two randomized clinical trials (AZA-001, and CALGB 9221) conducted in HR-MDS patients (6). AZA-001 improved survival in HR-MDS patients compared to standard care irrespective of age, karyotype, or bone marrow (BM) blast percentage, and was associated with increased time to AML progression and reduced requirement for red blood cell transfusion (7). In the CALGB 9221 study, AZA treated patients exhibited slower rates of leukemic transformation, with no differences in survival (8). In addition, the Spanish Group of Myelodysplastic Syndromes showed a lack of improvement in survival in unselected HR patients (9). AZA treatment induces hematological improvement in only 30-40% of treated patients, with most experiencing disease progression on therapy (10). These clinical experiences highlight the need to better understand the mechanisms mediating patient responses to AZA and to identify predictors of response.

The objective of our study was to investigate whether mutational or transcriptional profiles from MDS HPSCs can predict treatment responses. We hypothesized that mutational or transcriptional profiles predictive of treatment response to AZA would be revealed by comparing paired pre-/post AZA MDS samples. We found that somatic mutational burden was reduced following therapy in patients who responded to therapy. In pre-treatment samples, patients who showed reduced blast count were enriched in genes related to cell cycle, while blasts non-responders were enriched for pathways related to stemness. Interestingly, these same pathways did not predict response based on transcriptional profiles generated from post-AZA samples. A transcriptional classifier developed based on comparing gene expression between blast responders and non-responders to age-matched controls could predict patient response to therapy and overall survival in two independent MDS patient cohorts. Overall, these studies provide insights into the molecular mechanisms mediating AZA response and describe a novel transcriptional classifier that can predict survival of MDS patients at diagnosis.

## 2 Methods

### 2.1 Patient and control samples

Bone marrow (BM) samples from fourteen patients with MDS (n=11), and MDS transformed to AML (n=3) were collected before and during their treatment with AZA. Patients were diagnosed following the WHO 2017 criteria. Patient median age was 70 years (range: 49-85 years). MDS/AML patient samples were grouped into responder (R) and non-responder (NR) groups based on: i) reduction in the percentage of blasts (Blast Response), or ii) improvement of cytopenias (Hematological Response). Hematological evaluation of AZA response was performed according to the International Working Group (IWG) criteria for MDS and AML (11). Five out of fourteen patients were blast responders (BL-R), seven were blast non-responders (BL-NR), and for the remaining two patients, blast response could not be determined. When classifying patients by hematological response, eight of the fourteen patients were responders (HEM-R), four patients were non-responders (HEM-NR), and for two patients, hematological response could not be determined (different patients than the ones with undetermined blast response). Among the fourteen patients, three patients showed both BL-R and HEM-R, while three other patients were both BL-NR and HEM-NR. Baseline patient characteristics are shown in **Table 1**, and further detailed in **Supplementary Table 1**.

**Table 1:** Clinical characteristics of MDS patients included in the study.

Characteristics	Median (range)
Age (years)	70 (49-85)
Sex (n,%):	
Male/Female:	6 (42.9%)/8 (57.1%)
Blood cells count basal:	
Platelets (x 10^3^/mm^3^)	138.6 (9-470)
White blood cells (x 10^3^/mm^3^)	5.8 (1.2-36.6)
Hemoglobin (g/dL)	10.2 (7.2-14.9)
Absolute Neutrophil Count (ANC) (10^9^/L)	5.8 (1.2-36.6)
Bone Marrow blast (%)	13 (0.5-70)
IPSS-R Category:	
High/Intermediate	9/2
Diagnosis (n, %)	
MDS-EB-1	4, 28.6%
MDS-EB-2	5, 35.7%
MDS-MLD	2, 14.3%
AML-MRC	3, 21.4%
Type of Response (n, %)	
Blast/Hematological	5 (35%)/8 (57%)

MDS-EB-1 (MDS with excess blasts, type 1) is characterized by 5-9% blasts in the bone marrow or 2-4% blasts in the blood; MDS-EB-2 (MDS with excess blasts, type 2) is characterized by 10-19% blasts in the bone marrow or 5-19% blasts in the blood; MDS-MLD, MDS with multilineage dysplasia; AML-MRC, Acute myeloid leukemia with myelodysplasia-related changes.

### 2.2 Cell separation

Bone marrow mononuclear cells (BMMCs) were isolated from BM samples using Ficoll-Paque Plus (GE Healthcare, Massachusetts, US) density gradients per manufacturer’s instructions. CD34+ cells were isolated by immunomagnetic separation using an autoMACs Pro Separator (Miltenyi Biotec GmbH, Bergisch Gladbach, Germany) following the manufacturer´s instructions.

### 2.3 Nucleic acid extraction

Sorted cells were snap-frozen and stored at -80°C. Total DNA and RNA were extracted using the RNeasy Micro Kit (Qiagen, Valencia, CA, USA) following the manufacturer’s protocol. RNA integrity was assessed on an Agilent 2100 Bioanalyzer (Agilent Technologies, Santa Clara, CA, USA) using the RNA 6000 Nano Assay kit. DNA and RNA quantity were assessed by qubit 2.0 (ThermoFisher Scientific, Massachusetts, U.S.A.) using a DNA double-strand kit or an RNA kit (high/broad sensitivity).

### 2.4 Next generation sequencing

#### 2.4.1 DNA-seq

A custom high throughput sequencing (HTS) panel was made using Design Studio (Illumina, California, U.S.) that included 3,259 probes targeting 1,740 regions including exons, splicing regions and untranslated regions for 117 genes associated with myeloid neoplasms (**Supplementary Table 2**); the final design covered ~500kb. Libraries were made following Nextera Rapid Capture Custom Enrichment (Illumina, San Diego, CA, USA) protocol. Sequencing was performed on a NextSeq 550 sequencer with a read length of 2 × 150 nucleotides. Variant allele frequency (VAF) cut-off was set at 5% except for 2 specific variants in *NRAS* and *KRAS* genes in pre-treatment samples (VAFs 1.27 and 1.83, respectively), for which we detected significantly higher VAFs in post-treatment samples (15.29 and7.19,repectively), suggesting possible clonal expansion, and one variant in *TP53* detected with a VAF of 87.48 before treatment and 3.98 after treatment.

#### 2.4.2 RNA-seq

Libraries were prepared using the SMART-Seq v4 Ultra Low Input RNA kit (Clontech, California, U.S.), and sequenced on a HiSeq400 sequencer (Illumina) according to manufacturer´s protocol with a read length of 2 × 150 nucleotides. Following demultiplexing, data quality control, alignment, and generation of a read count matrix, DESeq2 was used for differential gene expression analysis.

### 2.5 Patient sample scoring in independent data cohorts

We assigned a score to each patient sample in the Pellagatti et al. cohort (12), based on the expression of the responder (R) and non-responder (NR) specific genes (compared to healthy controls), and further ranked the patients based on the Δ(NR-R) score. Patient samples with Δ(NR-R) ≥ median(Δ(NR-R)) were classified as NR-*like*, and samples with Δ(NR-R) <median(Δ(NR-R)) were classified as R-*like*. We used similar scoring strategy to re-classify MDS samples in a 2nd MDS cohort, GSE77750 (13).

### 2.6 Pathway analysis and data visualization

GSEA software package from the Broad Institute (http://www.broad.mit.edu/gsea/) was utilized to analyze enriched biological pathways in patient samples compared to control. R package ggplot2 (https://ggplot2.tidyverse.org.) was utilized to visualize the data.

### 2.7 Statistical analysis

To analyze the correlation between VAF from BMMC and CD34+ cells, a Pearson correlation coefficient was calculated to determine the strength and direction of their relationship. For investigating the association of different clinical parameters (e.g.MDS morphologic subtype, IPSS-R score) with transcriptional response signatures from publicly available MDS datasets, Pearson’s Chi-squared test with Yates’ continuity correction was implemented in R. For statistical analyses, P values were determined by applying the two-tailed t test for independent samples. All values were expressed as means ± SEM, with significance levels indicated as follows: *, P < 0.05; **, P < 0.005; and ***, P < 0.001. Analyses were performed using GraphPad Prism software.

## 3 Results

### 3.1 AZA alters mutational profiles in MDS

**Figure 1:**
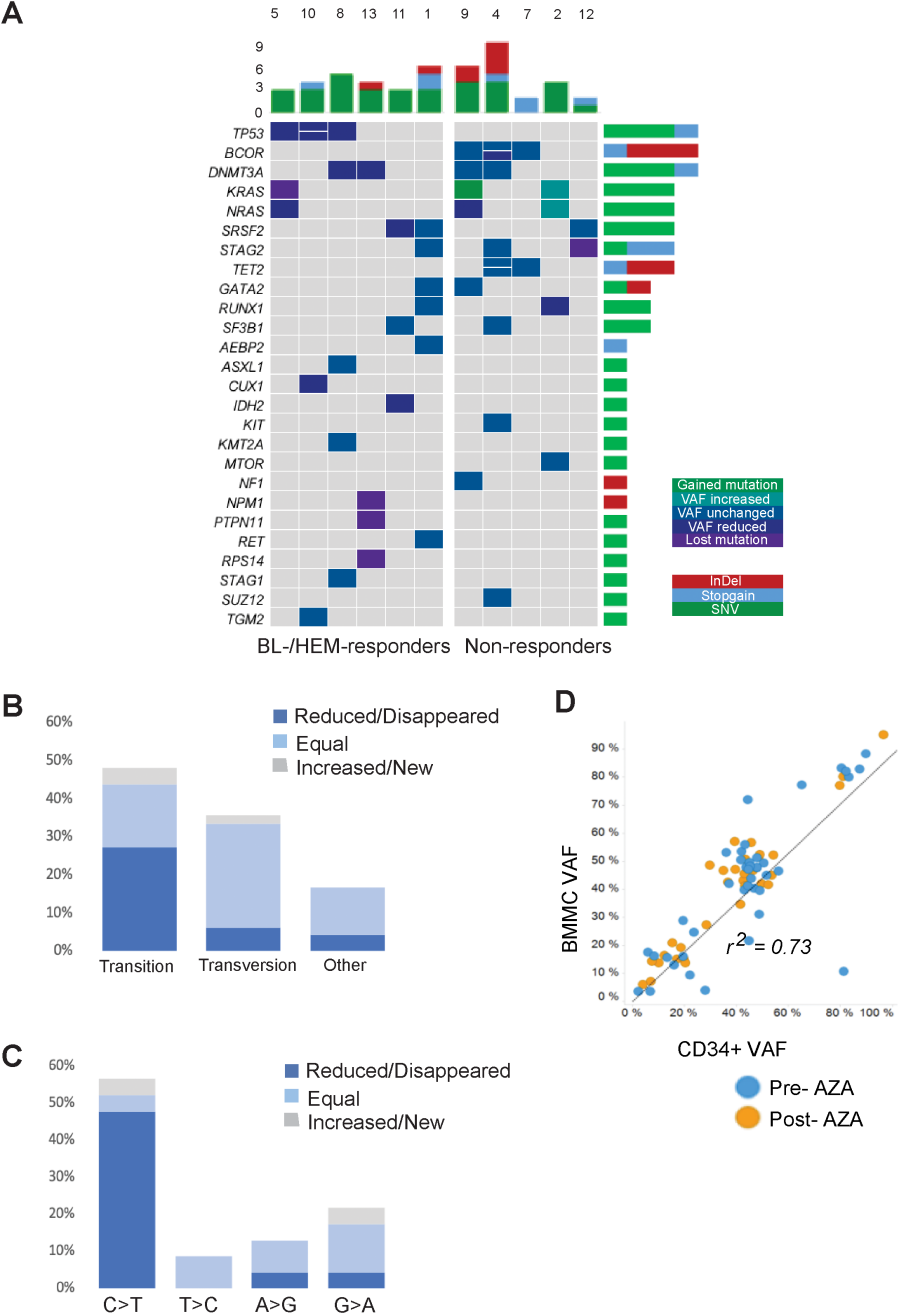
Comprehensive landscape of mutations in the MDS cohort. **(A)** Dynamics of gene mutations in CD34+ cells during AZA treatment. Genes are represented in rows; each column represents a patient. Dynamics are represented by a color gradient: green/cyan for newly acquire/increasing mutations, cobalt blue for stable mutations, and navy/violent colors for decreasing/disappearing mutations. **(B)** Proportion of transitions and transversions and how they change after AZA treatment. **(C)** Graphical representation of transition mutations and how they change after AZA treatment. **(D)** Ratio between VAF from BMMCs and CD34+ population. Blue plots represent mutations before AZA treatment and orange plots represent mutations after therapy.

Mutations were identified in eleven out of twelve patients (91.6%). A total of 48 mutations and a median of 4 mutations [range 0-9] per patient were observed. A detailed description of all the mutations is provided in **Supplementary Table 3**. AZA treatment reduced the mutational allele burden in at least one gene, in eight out of eleven (72.7%) patients. This decrease was more prominent in R than in NR patients (**Figure 1A**). All patients with *TP53* mutations exhibited blast or hematological response (BL-R, HEM-R) and showed reductions in *TP53* VAF post-AZA. In contrast, patients with *BCOR* mutations did not exhibit either type of response, and 3 out of 4 *BCOR* mutations identified in three patients showed no change in VAFs. Two patients with *DNMT3A* p.R693H mutations showed VAF reduction and BL-R or HEM-R, while two patients with mutations in other regions of *DNMT3A* neither showed changes in VAF nor achieved either type of response. Patients with *KRAS* and *NRAS* mutations pre-AZA displayed variable responses. For example, patient 2 with mutations in *MTOR*, *RUNX1*, *KRAS*, and NRAS pre-AZA, showed increased *KRAS* and *NRAS* VAFs post-AZA, and did not achieve blast or hematological response. Patient 9 acquired an additional mutation in *KRAS* while the *NRAS* burden decreased post-AZA. This patient did not experience a reduction in blast count and remained stable. In contrast, patient 5 harbored mutations in *TP53*, *KRAS* and *NRAS* at diagnosis, and VAFs for all 3 mutations decreased or became undetectable following AZA, despite the fact that the patient did not show BL-R or HEM-R.

Nucleoside inhibitors such as AZA can alter the composition of the dNTP pool, thereby inducing DNA damage responses and cell death (14). To assess if AZA alters nucleotide substitutions due to an imbalanced dNTP pool, we measured the frequencies of transitions and transversions. Pre-AZA samples showed transition and transversion proportions of 47.9% and 35.4%, respectively (**Figure 1B**). Notably, AZA treatment led to a significantly greater VAF reduction in transitions than transversions. In contrast, 76.5% of transversions exhibited no change in VAF versus 34.8% of transitions (**Figure 1B**). Among transition mutations, C>T was the most frequent, with eight cases exhibiting decreased, three cases exhibiting loss, and two cases exhibiting gain or stable VAF (**Figure 1C**). To determine the contribution of malignant clones to mature hematopoietic cells, we compared VAFs of CD34+ cells and matched BMMCs. While there was a high VAF correlation between pre-/post- AZA samples for both cell types (r=0.73), BMMCs consistently showed slightly higher VAFs (**Figure 1D**).

### 3.2 AZA treatment induces widespread gene expression changes in MDS HSPCs

**Figure 2:**
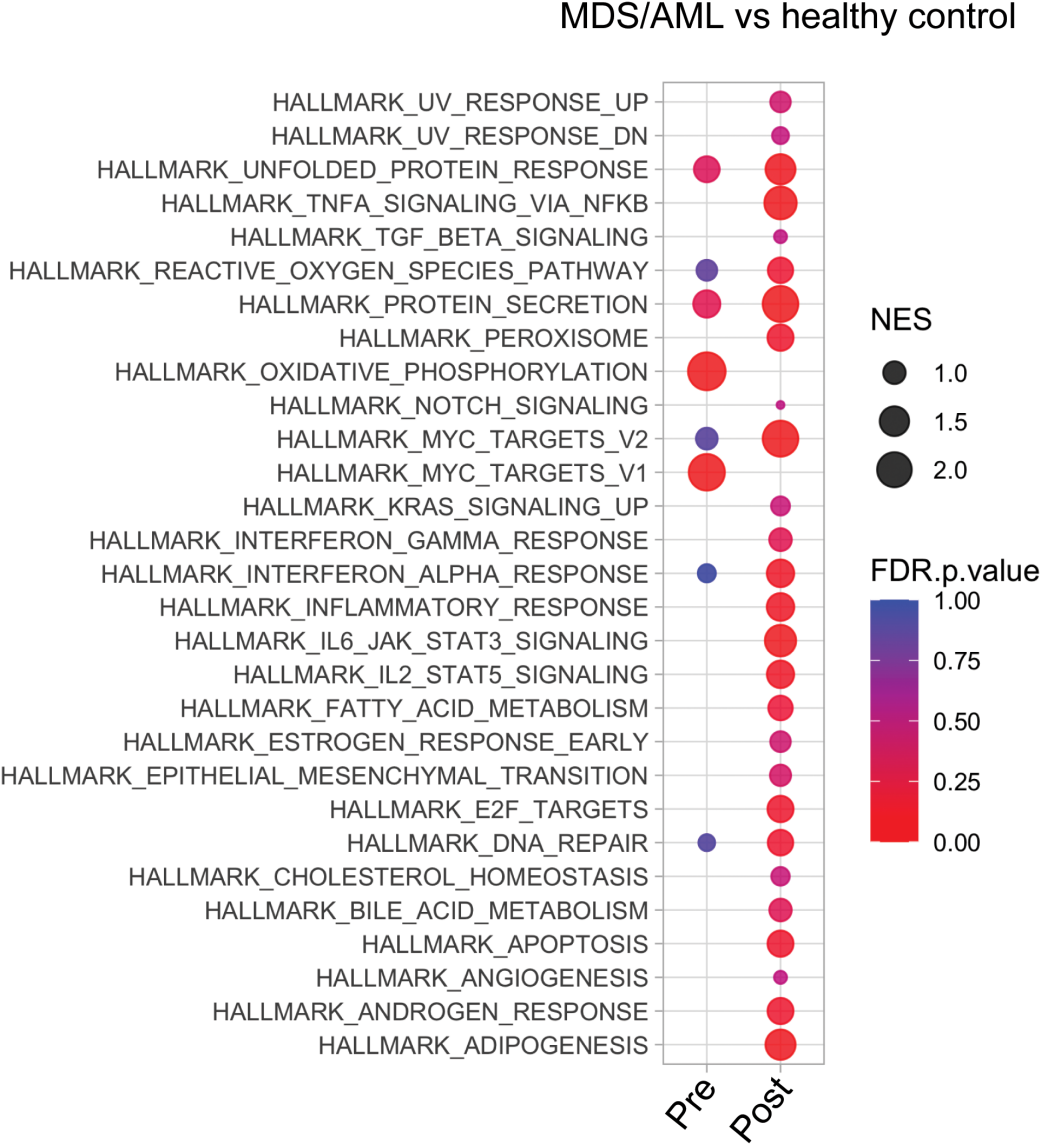
Transcriptional analysis of MDS HSPCs in comparison to healthy control CD34+ cells. Pathway analysis of pre- and post-treatment MDS HSPCs in comparison to healthy, age-matched controls.

We compared the transcriptional profiles of pre- and post-AZA MDS HSPCs with age-matched, healthy controls. Pre-treatment HSPCs exhibited 584 up-regulated genes and 1,040 down-regulated genes compared to controls (abs log2fold change (FC)≥1, p-value<0.01) (complete list in **Supplementary Table 4**). Post-AZA treatment samples showed 270 up-regulated and 1,230 down-regulated genes (abs log2fold change (FC)≥1, p-value<0.01) compared to controls (complete list in **Supplementary Table 5**). Pathways significantly enriched in pre-AZA patient HSPCs included oxidative phosphorylation and MYC targets. Post AZA treatment, all of these pathways lost enrichment, while pathways associated with unfolded protein response, secretion, reactive oxygen, interferon alpha, cell cycle, and DNA repair were enriched (**Figure 2**).

### 3.3 Gene signatures at diagnosis differ based on AZA response type

#### 3.3.1 Blast response

**Figure 3:**
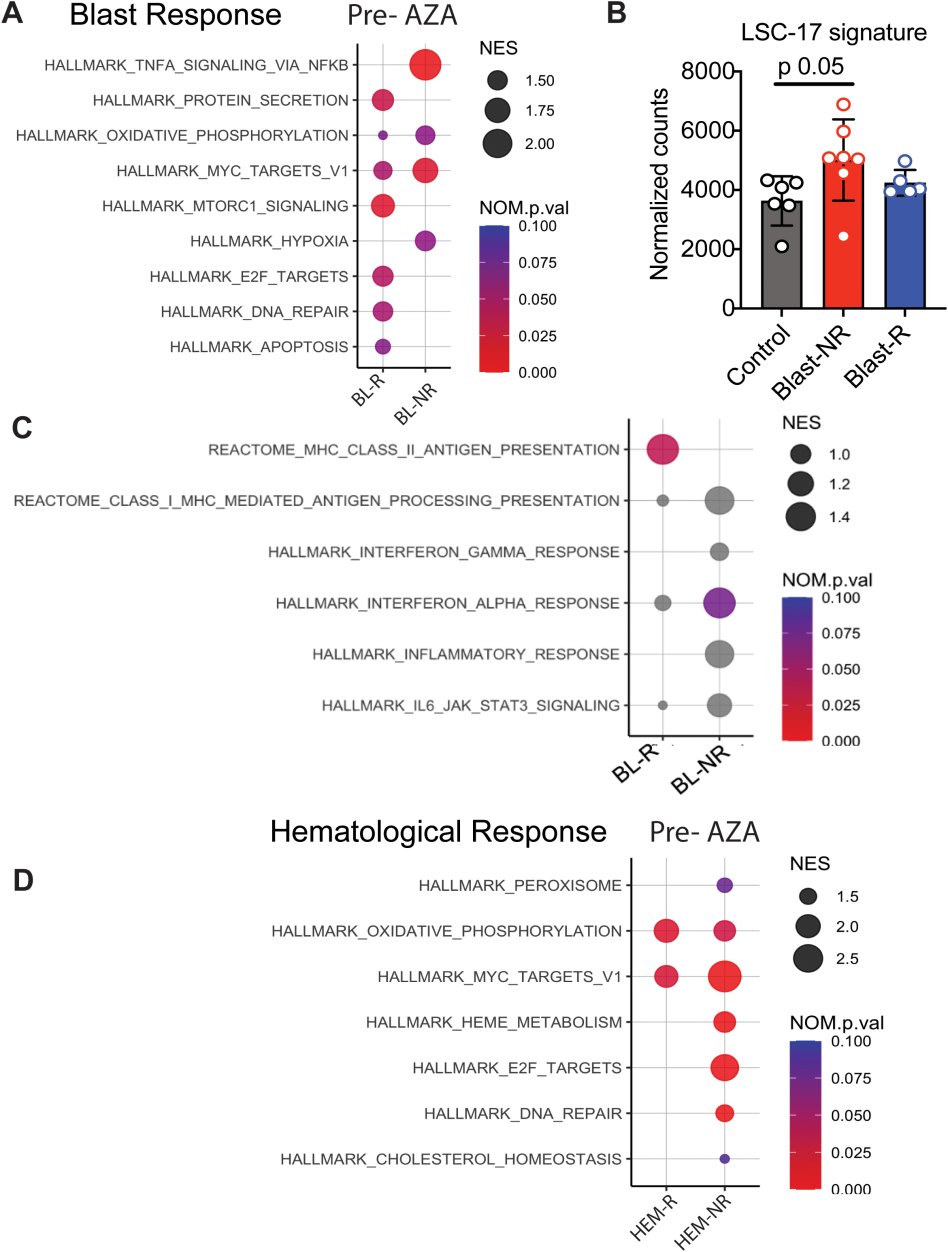
Transcriptional profiles of MDS patients associated with blast response before AZA treatment. **(A)** Pathway analysis of blast responders (BL-R) and non-responders (BL-NRs) before treatment with AZA. **(B)** Expression of LSC-17 genes in MDS HSPCs, based on BL-R and BL-NR gene signature. **(C)** Immunological pathway analysis of BL-R and BL-NRs before treatment with AZA. **(D)**. Pathway analysis of hematological responders (HEM-R) and non-responders (HEM-NRs) before treatment with AZA.

To identify pathways that might determine blast response, we identified the differentially expressed genes (DEGs) in pre-treatment samples of blast responder (BL-R) and blast non-responder (BL-NR) patients compared to age-matched controls. Genes up-regulated in BL-R patients were enriched in pathways such as protein secretion, MTOR signaling, E2F targets, DNA repair, and apoptosis, consistent with an enrichment of cycling cells. In contrast, genes up-regulated in BL-NR patients were enriched for pathways such as TNFa signaling via NFkB and MYC target genes (**Figure 3A**). TNFa signaling has been shown to promote HSC renewal and myeloid-biased regeneration (15) as well as leukemia-initiating cell capacity in AML (16). MYC is a well-known driver of AML pathogenesis and promotes leukemia stem cell (LSC) self-renewal and chemotherapy resistance (17–19). Since these data suggest that AZA resistance is due to enrichment for LSC transcriptional programs, we assessed the expression of LSC genes (20). BL-NR patients showed increased expression of these genes compared to BL-R patients and controls (**Figure 3B**).

Since the immune system surveils neoplastic HSPCs in MDS/AML (21), we next investigated whether HSPCs in MDS exhibit evidence of cell-intrinsic differences in immune recognition. Notably, BL-R samples were enriched for genes associated with major histocompatibility class II (MHC class II) presentation, while BL-NR samples did not exhibit enrichment of Hallmark immunological pathways, including inflammation-associated and interferon-associated genes (**Figure 3C**).

#### 3.3.2 Hematological response

Given that hematological and blast responses may depend on distinct molecular mechanisms, we separately investigated samples based on hematological response. Prior to therapy, hematological responder (HEM-R) and hematological non-responder (HEM-NR) patient samples were enriched in pathways such as oxidative phosphorylation and MYC target genes compared to controls, but HEM-NR samples were uniquely enriched for genes present in the E2F target, DNA repair, and heme metabolism signatures (**Figure 3D**).

### 3.4 AZA induces similar transcriptional signatures regardless of treatment response

In order to evaluate MDS HSPC responses to AZA treatment, we compared the transcriptional profiles of paired pre-/post-AZA samples.

#### 3.4.1 Blast response

**Figure 4:**
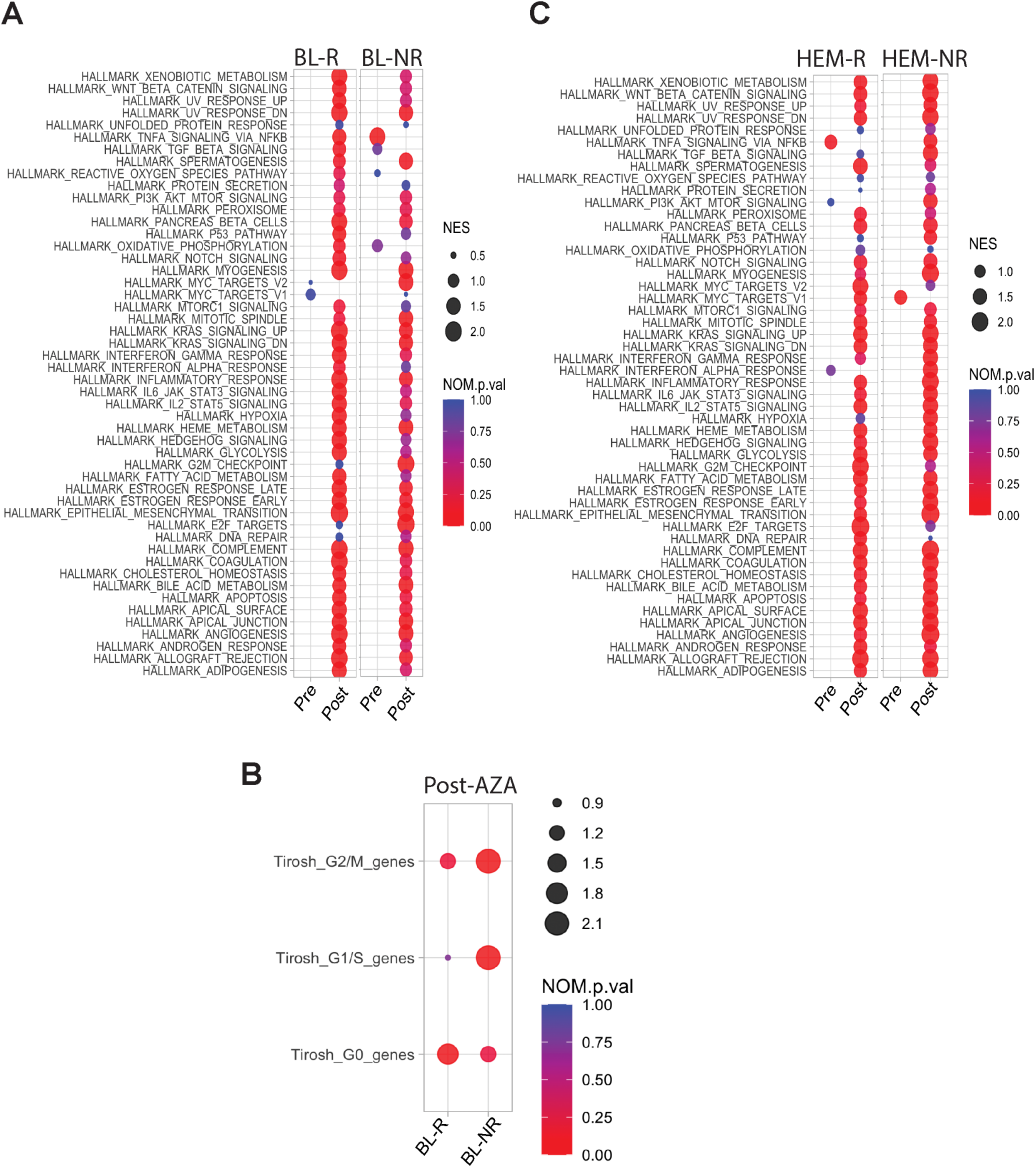
Transcriptional profiles of pre-/post-AZA treatment MDS HSPCs. **(A)** Pathways enriched in BL-R and BL-NR samples pre-/post-AZA **(B)** Cell cycle associated pathways in BL-R and BL-NR samples pre-/post-AZA **(C)** Pathways enriched in HEM-R and HEM-NR samples pre-/post-AZA.

Fewer DEGs were identified in post- *vs* pre-AZA specimens in BL-R than in BL-NR patients (1,810 *vs* 2,300, respectively; complete lists in **Supplementary Tables 6, 7**). Despite these differences, AZA induced enrichment of almost all Hallmark pathways including cell cycle changes in both BL-R and BL-NR groups, similar to prior studies (22). A notable exception was the TNFa pathway, which was significantly enriched in pre-AZA BL-NR samples. Compared to pre-treatment samples, post-AZA BL-R patients showed enrichment of MYC targets, E2F targets, and DNA repair pathways (**Figure 4A**), and post-AZA BL-NR samples showed significant expression of cell cycle genes (**Figure 4B**).

#### 3.4.2 Hematological response

Post-AZA, more DEGs were identified in HEM-NR than in HEM-R patients (3,202 genes vs 1,570 genes, respectively) (complete lists in **Supplementary Tables 8, 9**). Pathway analysis showed significant enrichment of the TNFa signaling via NFkB pathway in pre-AZA HEM-R patients, which was lost upon AZA treatment (**Figure 4C**). Pre-AZA HEM-NR samples showed enrichment of MYC target genes (**Figure 4C**). Analysis of cell cycle gene expression changes showed that AZA induces cell cycle genes irrespective of hematological response type (**Supplementary Figure 1A**).

### 3.5 Blast response to AZA is associated with specific transcriptional programs

To evaluate potential AZA induced changes in differentiation, we performed a CIBERSORTx analysis using established gene expression profiles from healthy HSPC populations (23). Comparisons of pre- and post-AZA samples revealed no significant changes among the HSPC populations assessed (**Supplementary Figure 1B**). Early (low-risk) MDS shows reduced frequencies of immunophenotypically defined B-cell progenitors (24). While pre-AZA HSPCs showed down-regulation of B-lymphocyte genes (43 out of 56 genes) compared to healthy controls (**Table 2**; **Supplementary Table 10**), CIBERSORTx analysis predicted negligible numbers of common lymphoid progenitors (CLP) in patient samples. After AZA treatment, specific B-cell development genes were induced, with 30 genes up-regulated in post-AZA vs pre-AZA, but CIBERSORTx did not predict a restoration of CLP numbers. While BL-R samples showed a trend toward up-regulation of B-cell development genes compared to BL-NR samples, no correlation was found between hematological response and induction of B-cell genes.

**Table 2:** Average expression of B-cell development genes in healthy controls, pre- and post-AZA treatment in blast responder (BL-R) and non-responder (BL-NR) patients.

Gene	Control	BL-R Pre	BL-R Post	BL-NR Pre	BL-NR Post
IGHG3	16104.79	9435.45	82198.04	1648.41	7598.11
IGHM	88968.79	28365.72	62260.41	36947.57	43268.96
CD14	5778.63	1715.27	12184.44	3109.39	3771.74
FABP4	8830.79	776.97	7072.81	3947.00	3899.05
CRHBP	5048.48	1780.38	3809.01	5571.75	8213.73
CD24	4065.58	421.05	3622.90	239.80	1635.12
LPL	1425.19	269.02	1783.08	657.64	3369.90
RGS1	3628.81	1542.55	5145.90	4721.61	4405.77
NNMT	942.07	194.09	1004.29	529.55	2819.85
EPAS1	1703.03	227.54	1799.50	780.56	1646.70
LTB	4088.90	2072.56	2084.41	703.23	2654.48
CD79B	14484.97	2412.57	1666.90	1515.04	4032.88
COL5A1	5024.98	1167.26	1017.97	778.64	1940.60
CAV1	312.86	223.45	1187.46	562.42	610.42
LEF1	5425.96	697.48	627.26	79.38	1016.49
VPREB1	8653.12	838.01	377.65	118.67	1392.64
POU2AF1	3184.54	244.71	579.62	14.78	473.98
FCN1	33193.86	5406.90	19291.68	20524.88	7421.06
SPARCL1	1199.84	154.30	847.43	604.10	651.28
LRIG1	3738.12	479.57	537.37	459.50	1059.65
GEM	131.59	429.96	1000.10	513.93	312.95
BLNK	1970.59	1207.32	1492.85	270.21	297.82
RIMS3	1807.37	99.94	154.38	78.88	325.03
AKAP12	5949.82	549.22	1573.43	2254.97	1480.03
QRSL1	2735.24	2127.55	2005.38	1591.73	1959.36
STAG3	876.34	550.38	560.50	188.22	378.26
P4HA2	576.43	135.73	152.42	164.42	323.24
VPREB3	3576.61	287.05	58.75	15.50	378.48
CRIM1	1080.04	389.77	481.16	943.00	909.27
PAX5	1693.54	36.27	6.42	4.14	68.51
BACH2	2928.52	250.45	162.96	281.25	354.82
CCL20	22.60	25.58	32.13	100.11	60.63
DNTT	46819.78	10225.85	7005.71	2725.81	5589.69
MME	5154.99	238.26	198.33	1113.70	782.56
HES1	381.46	571.60	276.11	436.19	296.29
ISG20	3444.08	1371.75	1766.16	2678.35	1827.40
ID3	1957.32	1599.76	998.57	1026.62	1153.00
IGLL1	25769.20	15318.90	12693.64	5020.73	7141.67
SSBP2	6413.97	4926.93	4279.38	5677.61	5791.00
SKIL	3087.93	2242.85	1847.12	1960.56	1805.89
C6orf62	8376.06	7788.41	9180.92	9785.68	7653.82
HLA-DQA1	2865.25	1941.78	3661.78	4099.04	1635.60
PPFIBP1	1055.90	1245.71	761.11	1464.38	1015.21
CXCR4	24631.48	11656.08	16431.12	23169.72	17397.25
NR4A3	1000.55	348.15	605.17	2596.13	611.65
HSPA1B	1005.70	1081.68	1386.80	3446.61	1355.19
ID1	2345.93	3871.06	2496.06	3319.61	2543.26
CD9	8324.22	3264.77	4757.83	10320.21	6274.36
CDK9	11139.21	6832.92	4588.04	6137.37	5560.83
HLA-DQB1	9128.92	4356.27	6704.73	11541.73	5338.77
BTG1	21182.78	6520.01	9899.51	18182.66	10877.76
PDE4B	6622.82	6806.24	8811.91	14083.89	7035.40
NR4A1	5499.83	10022.47	10095.90	14649.17	9185.77
NR4A2	5204.74	3324.52	5709.50	16996.50	5647.31
TCF4	15473.58	10846.44	7542.07	15785.89	7603.98
ZFP36L2	263541.21	113923.29	84431.10	144233.36	152912.72

### 3.6 Blast response gene signature can predict AZA response and overall survival

**Figure 5:**
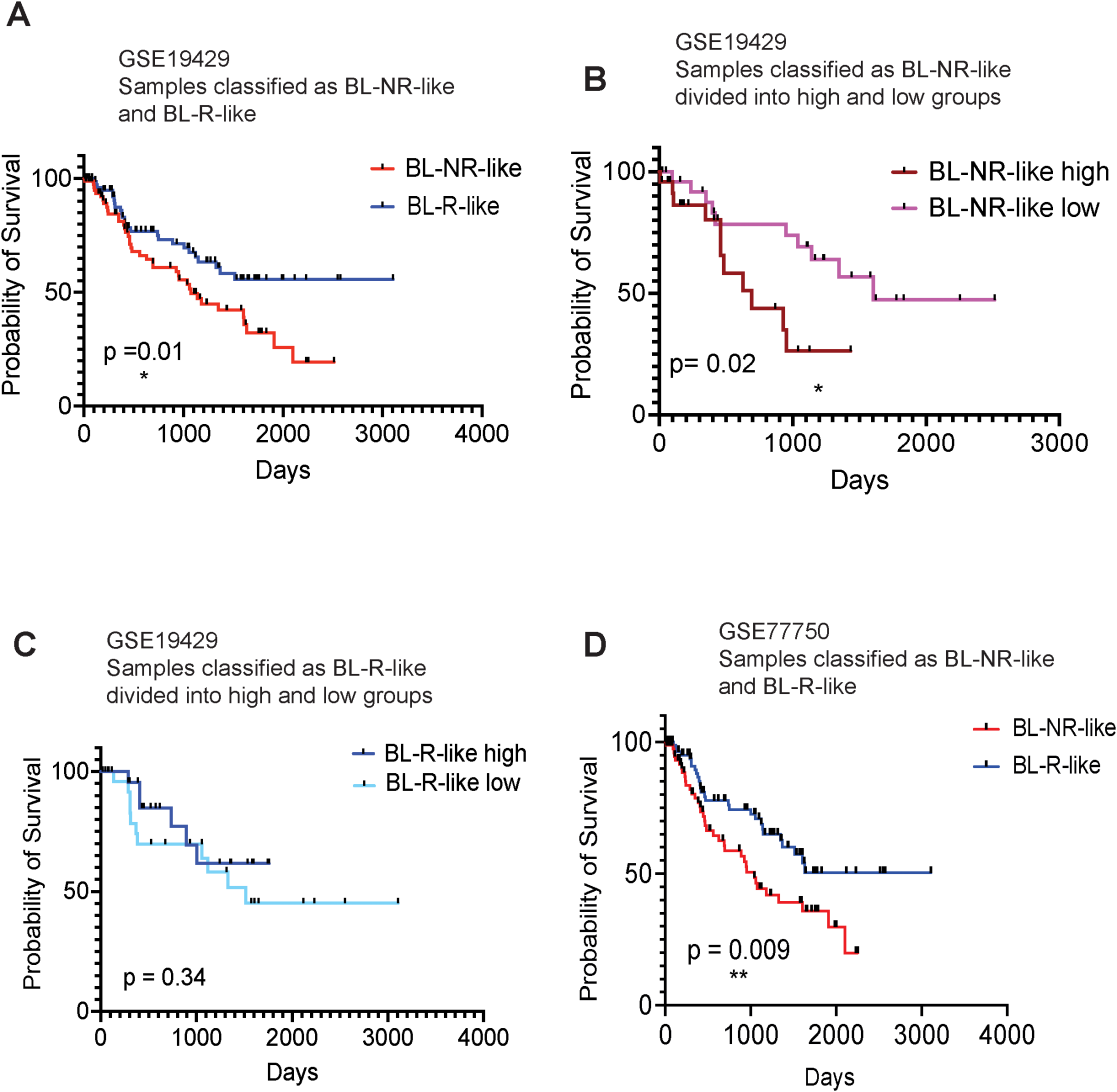
Kaplan-Meier survival analysis of MDS patients, based on BL-R and BL-NR gene signature **(A)** Survival analysis of patients, recorded in GSE19429, based on sample classification into blast R-*like*, or NR-*like*, according to our blast-R and blast-NR gene signature. **(B)** Survival analysis of NR-*like* patients from **(A)**, based on high, or low expression of blast-NR signature. **(C)** Survival analysis of R-like patients from **(A)**, based on high, or low expression of blast-R signature. **(D)** Survival analysis of patients recorded in GSE77750, classified as blast-R-like or NR-*like*, based on our blast-R and blast-NR gene signature.

To determine whether gene signatures associated with blast response may predict MDS/AML patient outcomes, we first generated gene expression profiles for the pre-AZA BL-R and BL-NR groups by identifying genes significantly and uniquely expressed in each group compared to healthy, age-matched controls. BL-R and BL-NR groups comprised 1,292 and 1,748 genes respectively. We then generated a score that would allow measurement of the similarity of any diagnostic MDS sample to the BL-R or BL-NR patients in our cohort, and enable assignment of each new patient sample to BL-R-*like* or BL-NR-*like* groups based on their transcriptional score. Utilizing this strategy to classify 183 MDS patients [GSE19429 (12)], we found that BL-NR-*like* patients showed significantly poorer survival than BL-R-*like* patients (**Figure 5A**). We further stratified the BL-NR-*like* patients into BL-NR-*like* ‘high’ and BL-NR-*like* ‘low’ groups. BL-NR-*like* ‘high’ patients showed significantly shorter survival compared to NR-*like* ‘low’ patients (**Figure 5B**). No significant differences in survival were observed between BL-R-*like* ‘high’ and BL-R-*like* ‘low’ groups, although BL-R-*like* ‘high’ patients showed a trend towards longer survival (**Figure 5C**). We evaluated the available clinical parameters for these two groups of patients, and found that compared to the BL-NR-like patients, BL-R-like patients were significantly enriched for patients in the very low IPSS score group (Pearson’s Chi-squared test with Yates’ continuity correction p-value =0.05) as well as in the refractory anemia with ring sideroblasts (RARS) morphologic subtype of MDS (Pearson’s Chi-squared test with Yates’ continuity correction p-value =0.04) (**Supplementary Figure 2**). We also investigated the clinical parameters associated with 37 MDS patients re-classified as putative BL-NR-like and BL-R-like in a second data cohort [GSE111085 (25)], but did not find any significant association with any IPSS category or disease subtype (**Supplementary Figure 2**).

To further investigate the ability of our transcriptional classifier to predict survival, we examined the publicly available gene expression profiles of 22 MDS/AML patients (13 responders and 9 non-responders; GSE77750) (13). We applied our approach to derive independent BL-NR and BL-R gene signatures from this dataset and classified the MDS patients in (12) as BL-NR*-like* and BL-R*-like*. When the GSE77750-derived transcriptional classifier was used, BL-NR*-like* patients exhibited significantly worse outcomes than BL-R*-like* patients (**Figure 5D**). Of note, our prognostic transcriptional classifier outperformed the algorithm originally described in this study.

We next evaluated MDS patient outcomes in the patient cohort in ref (12). using the same strategy to develop a transcriptional classifier based on hematological response. HEM-NR-*like* patients did not exhibit significant differences in survival compared to the HEM-R-*like* patients (**Supplementary Figure 3**). Examination of clinical histories revealed that three of the HEM-R patients (P1, P10, P13) also were BL-NRs. Given the potential contribution of blast number to patient outcomes, we re-evaluated the cohort after excluding these 3 patients from the analysis. This analysis confirmed no difference in survival between HEM-R-*like* and HEM-NR-*like* patients (data not shown).

## 4 Discussion

For over a decade, AZA has been the standard of care for MDS. However, only ~50% of patients will respond, and even those who do, have a poor prognosis after initial response (4, 25). Furthermore, clinical outcomes for AZA refractory/relapsed patients are extremely poor (25, 26). We evaluated paired pre-/post-AZA samples to better understand the biological consequences and mechanisms of AZA therapy. We found that AZA reduced the mutational burden in the majority of patients, particularly in those who exhibited clinical response to therapy. Our limited data appear to confirm prior reports that AML/MDS patients harboring *TP53* and *DNMT3A* p.R696H mutations respond to HMA therapy (27, 28). 3 patients harboring mutations in *BCOR* mutations exhibited no response, suggesting its role in AZA resistance, as has been suggested by prior studies in AML (29). We also observed that transitions, especially C>T, predominated over transversions, consistent with previous studies (30). Interestingly, the VAF of transition mutations was reduced or disappeared in most cases, suggesting that the 5mC mutations may be reversed by AZA. Collectively, these findings highlight the mutation-specific alterations associated with AZA response.

Comparison of pre-AZA HSPCs to age-matched healthy controls revealed enrichment of several pathways that promote self-renewal of HSC and LSCs. However, following AZA treatment, MDS HSPCs showed widespread induction of pathways including those associated with inflammation, cell cycling, and apoptosis. To detect potential outliers in our sample cohort, we conducted single sample GSEA (ssGSEA), but did not identify clear outliers based on pathway differences (data not shown). Although these pathway changes were observed in all patients regardless of blast or hematological response, blast non-responder (BL-NR) patients showed a lack of enrichment for cell cycle-associated pathways pre-treatment, consistent with prior reports that AZA resistant HSPCs are more quiescent (25). Compared to blast responder (BL-R) patients and age-matched healthy controls, BL-NR patients exhibited increased expression of a clinically-relevant LSC- signature. Additionally, BL-NR HSPCs showed lower expression of genes associated with MHC class II presentation than BL-R HSPCs, indicating that differences in immune cell recognition and clearance may affect patient response to AZA therapy. This is consistent with prior studies demonstrating the importance of immune cell-mediated blast clearance in MDS and AZA enhancement of this activity (31–35). Collectively, these findings indicate that multiple cell-intrinsic differences contribute to AZA resistance in MDS HSPC.

While prior studies have attempted to develop transcriptional signatures that predict AZA response or survival, these efforts have not resulted in robust methods to predict outcomes (13, 25). In contrast to previous studies, we compared patient samples to healthy controls and refined gene sets by only considering genes associated with the presence or absence of response. Our method applied to one MDS patient cohort (13) outperformed the prognostic gene signature derived utilizing gene expression data from the same cohort.

While our studies suggest that a transcriptional classifier may be used to predict AZA responses, they only focused on genomic and transcriptomic changes following AZA treatment. We expect that an integrated evaluation of epigenetic and post-transcriptional states with mutational and transcriptional states associated with AZA resistance would increase our ability to predict patient responses to AZA.

## Data Availability

Processed files are available at GEO, accession no. GSE272173.

## Supplementary material

Supplementary Figure 1 Transcriptional analysis of MDS HSPCs. **(A)** Cell cycle associated pathways in HEM-R/-NR pre-/post-AZA. **(B)** Imputation of the progenitor population distribution within MDS/AML CD34+ cell population by CIBERSORTx analysis, pre- and post-AZA. We did not observe any statistically significant difference between any of the pre-/post- comparisons. Samples have been color coded by their BL, or HEM- response.

Supplementary Figure 2 Clinical information of the patient samples from GSE19429, and GSE111085, which were re-classified as BL-R-like and BL-NR-like.

Supplementary Figure 3 Kaplan-Meir survival analysis of MDS patients, based on HEM-R and HEM-NR gene signature. Survival analysis of MDS patients (data from GSE19429), based on sample classification into HEM-R-like, or HEM-NR-like, according to our HEM-R and HEM-NR gene signature.

Supplementary Table 1 Clinical and biological characteristics of the patients treated with 5-azacytidine.

Supplementary Table 2 Myeloid-related genes included in the NGS panel.

Supplementary Table 3 Description of all mutations presented in MDS HSPCs.

Supplementary Table 4 List of genes differentially expressed between pre-treatment MDS HSPCs and healthy controls.

Supplementary Table 5 List of genes differentially expressed between post-treatment MDS HSPCs and healthy controls.

Supplementary Table 6 List of genes differentially expressed between pre- and post-treatment blast responders (BL-R).

Supplementary Table 7 List of genes differentially expressed between pre- and post-treatment blast non-responders (BL-NR).

Supplementary Table 8 List of genes differentially expressed between pre- and post-treatment hematological responders (HEM-R).

Supplementary Table 9 List of genes differentially expressed between pre- and post-treatment hematological non-responders (HEM-NR).

Supplementary Table 10 List of B-lymphocyte genes expressed in pre- and post-treatment patient samples and healthy controls.
