# Supplementary figures and images for: Molecular profiling of pre- and post- 5-azacytidine myelodysplastic syndrome samples identifies predictors of response
Source: Front Oncol. 2024 Sep 23;14:1438052. doi: 10.3389/fonc.2024.1438052 (PMC11456566; doi:10.3389/fonc.2024.1438052)

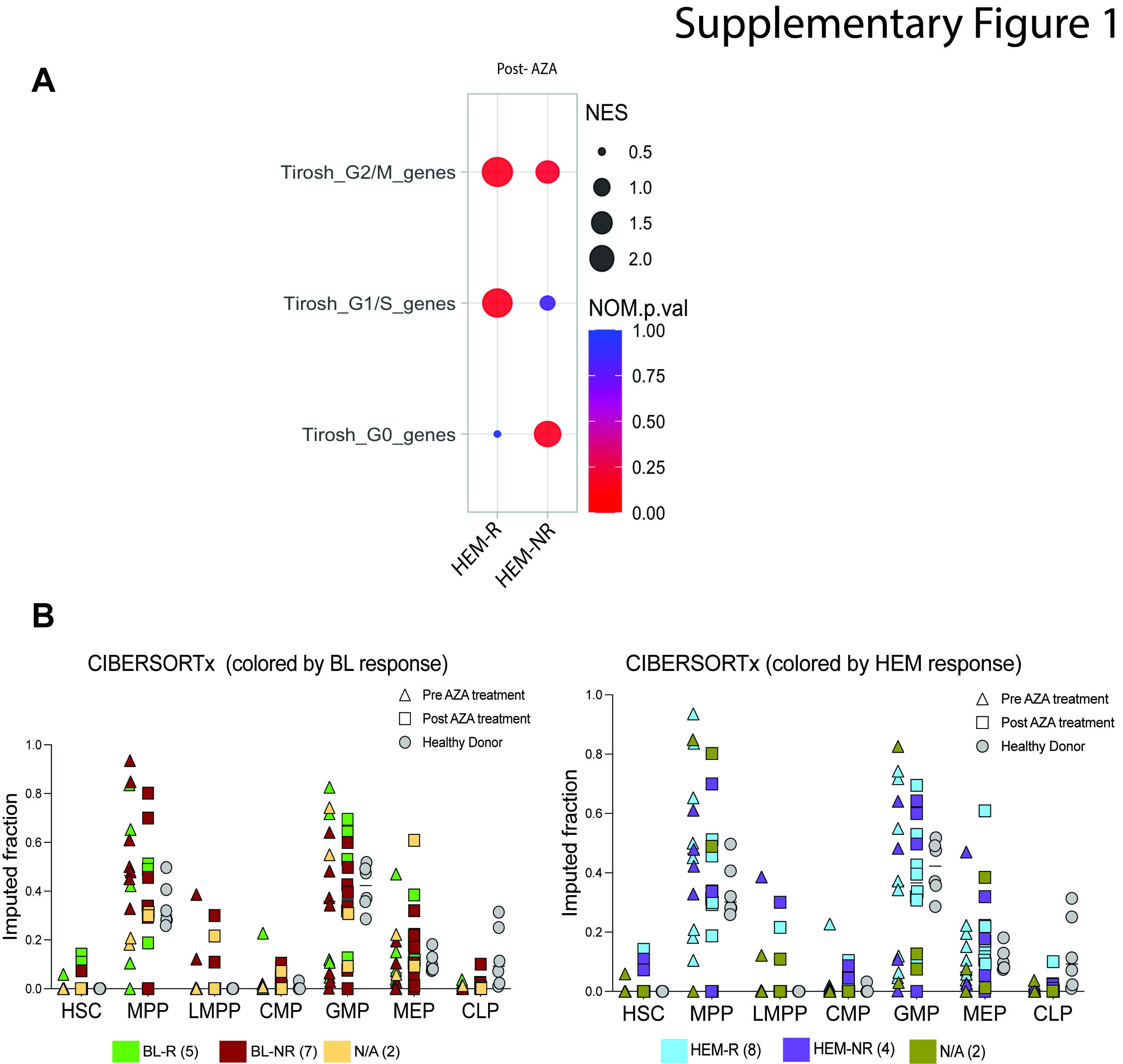

Supplement: Supplementary Figure 1 — Transcriptional analysis of MDS HSPCs. (A) Cell cycle associated pathways in HEM-R/-NR pre-/post-AZA. (B) Imputation of the progenitor population distribution within MDS/AML CD34+ cell population by CIBERSORTx analysis, pre- and post-AZA. We did not observe any statistically significant difference between any of the pre-/post- comparisons. Samples have been color coded by their BL, or HEM- response. [file Image1.tif]

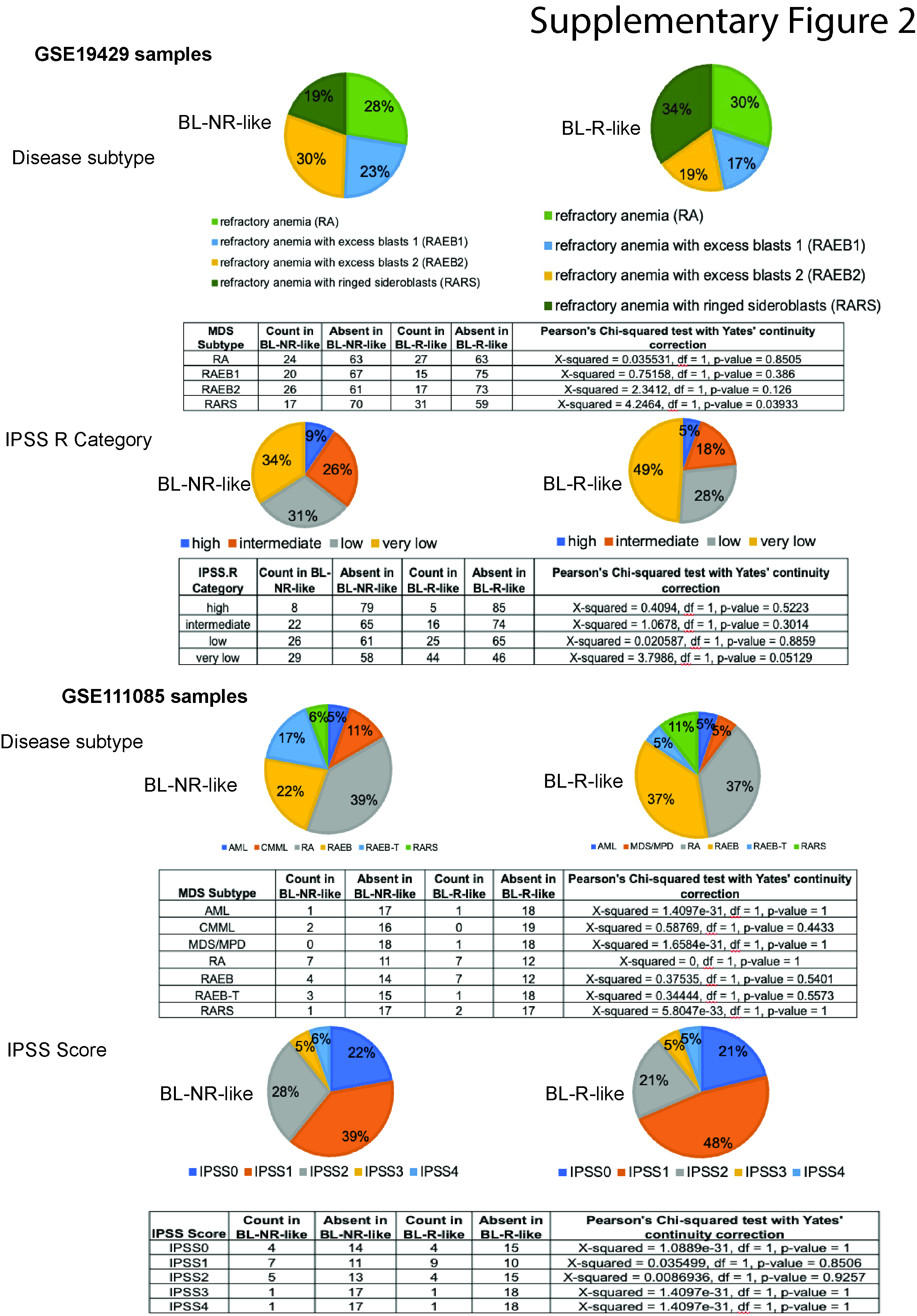

Supplement: Supplementary Figure 2 — Clinical information of the patient samples from GSE19429, and GSE111085, which were re-classified as BL-R-like and BL-NR-like. [file Image2.tif]

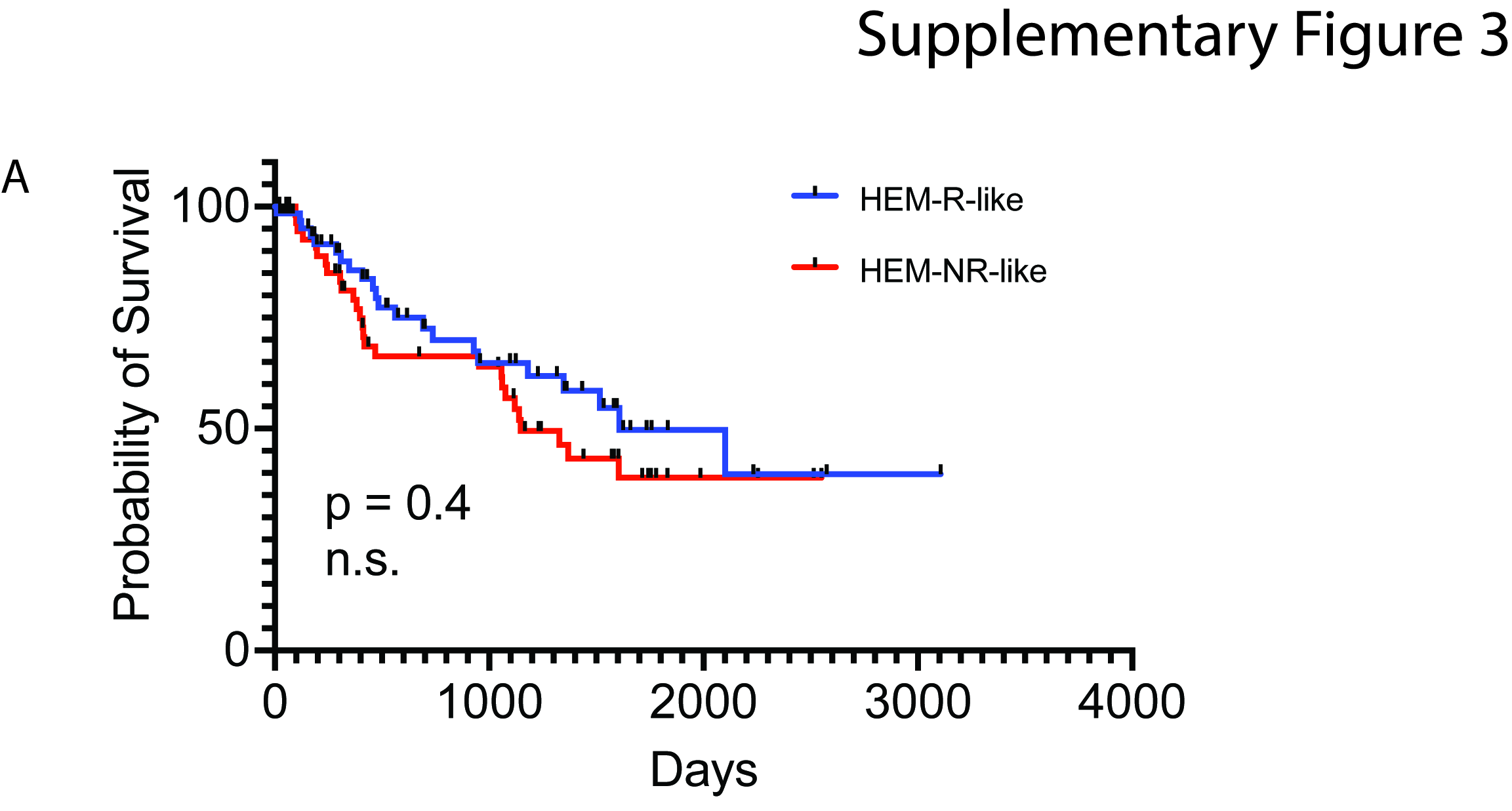

Supplement: Supplementary Figure 3 — Kaplan-Meir survival analysis of MDS patients, based on HEM-R and HEM-NR gene signature. Survival analysis of MDS patients (data from GSE19429), based on sample classification into HEM-R-like, or HEM-NR-like, according to our HEM-R and HEM-NR gene signature. [file Image3.tif]
